# Case Report: Surgical management of mandibular oral histiocytic sarcoma in a ragdoll cat with favorable outcome

**DOI:** 10.3389/fvets.2026.1880267

**Published:** 2026-08-12

**Authors:** Byung-Joon Seung, Jongmun Cho, Byungho Lee, Jungwhan Chon, Kihoon Kim

**Affiliations:** 1 Department of Pathology, College of Medicine, Kangwon National University, Chuncheon, Republic of Korea; 2 Haedeun Animal Medical Center, Yangju, Republic of Korea; 3 Department of Food Science and Biotechnology, College of Biotechnology and Bioscience, Kangwon National University, Chuncheon, Republic of Korea; 4 Department of Veterinary Surgery, College of Veterinary Medicine, Kangwon National University, Chuncheon, Republic of Korea

**Keywords:** feline, histiocytic sarcoma, mandibular tumor, oral tumor, rostral mandibulectomy

## Abstract

Feline oral histiocytic sarcoma (HS) is an exceptionally rare malignancy with limited clinical characterization and a poor prognosis. This report describes a case of mandibular HS in a 6-year-old Ragdoll cat that achieved a favorable local control following surgical management alone. The cat presented with left mandibular swelling, and diagnostic imaging revealed a poorly demarcated mass with extensive osteolysis of the mandible. Histopathological and immunohistochemical findings supported the diagnosis of a poorly differentiated malignant mesenchymal neoplasm with histiocytic differentiation. Rostral mandibulectomy with 0.5 cm surgical margins was performed. Postoperative recovery was uneventful, and no adjuvant chemotherapy was administered owing to financial constraints. The patient presented with clinical signs of lethargy and anorexia 7 months after surgery. On thoracic radiography, several soft tissue opacity masses throughout the bilateral lung lobes were identified. On ultrasonographic examination, multiple masses were also identified in the liver and kidneys, raising suspicion for metastatic lesion. However, there was no evidence of recurrence at the primary surgical site. The patient was euthanized. Compared with previously reported cases that showed poor outcomes despite additional therapies, this case suggests that surgical resection may provide successful local control in select cases. This report expands the clinical spectrum of feline oral HS and highlights the potential role of surgical excision as a primary treatment.

## 1 Introduction

Malignant oral neoplasms in cats are locally invasive and often associated with an unfavorable prognosis (1, 2). Squamous cell carcinoma is the most commonly reported oral neoplasm in cats, followed by mesenchymal tumors such as fibrosarcoma (3–5). Histiocytic tumors of the oral cavity have rarely been reported in cats (6, 7).

Histiocytic sarcoma (HS) is a malignant neoplasm of the dendritic cell or macrophage lineage, characterized by rapid dissemination and a generally poor prognosis (8). In dogs, primary lesions most commonly arise in the spleen, lymph nodes, lungs, bone marrow, skin, and periarticular tissues (8, 9). Histiocytic sarcomas are much less common in cats than in dogs. Feline histiocytic disorders include progressive histiocytosis, which typically begins as a cutaneous disease and may later involve internal organs, as well as disseminated and hemophagocytic forms of histiocytic sarcoma (8, 10). Primary oral HS is an exceptionally rare manifestation, with only two cases reported to date (6, 7).

Herein, we report a case of mandibular gingival HS in a relatively young (6-year-old) Ragdoll cat. Unlike previously reported cases involving older cats that survived for no longer than 6 months, even with additional treatment, the present case achieved survival for more than 6 months following surgical treatment without chemotherapy. Herein, we report this case to broaden our understanding of the clinical diversity of oral HS in cats.

## 2 Case description

A 6-year-old castrated male Ragdoll cat weighing 5.2 kg was referred for the evaluation of left mandibular swelling. Physical examination revealed a mass, and the left mandible was markedly swollen. On blood examination, complete blood count, serum biochemical analysis, and blood gas and electrolyte evaluations were within normal limits. Thoracic radiographic findings were unremarkable.

**Figure 1:**
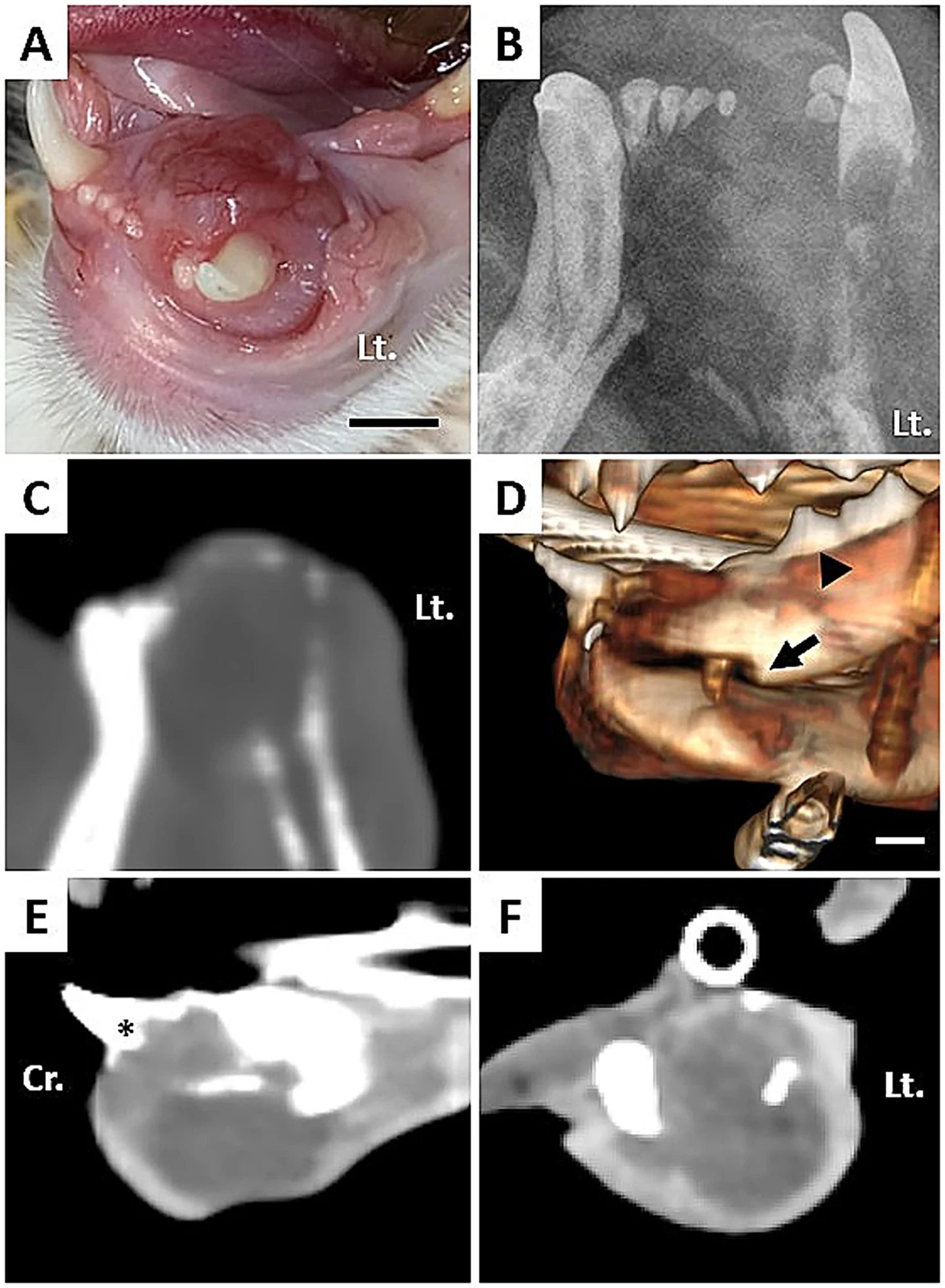
**(A)** Gross appearance of the mass at the time of presentation (Lt.: left). The mass was identified cranial to the lingual frenulum, between the canine teeth, with a left-sided predominance (Scale bar = 1 cm). **(B)** On dental radiographic examination, diffuse mandibular osteolysis extending bilaterally, with marked resorption of the left mandibular canine and incisor teeth and widespread tooth resorptive lesions up to the third premolars, was observed. **(C)** Bone window CT dorsal multiplanar reconstruction image demonstrating the extent of mandibular bone destruction. **(D)** Three-dimensional volume-rendered CT image demonstrating the extent of mandibular bone destruction. The caudal margin of osteolytic lesion (arrow) extended to approximately 0.5 cm mesial to the left second premolar tooth (arrowhead) (Scale bar = 0.5 cm). **(E)** Post-contrast sagittal CT image. Extensive osteolysis of the mandible extending to the level of the apex of the right canine tooth (asterisk) and invasion of the surrounding soft tissues (Cr.: cranial). **(F)** Post-contrast CT image. A mass with heterogeneous contrast enhancement without a distinct capsule was identified.

A thorough gross examination of the oral cavity was performed under general anesthesia. A 30 × 25 × 30 mm firm, ulcerated, sessile mass arising from the oral mucosa rostral to the lingual frenulum, with a left-sided predominance, was identified (Figure 1A). Diffuse osteolysis of the mandible was identified on dental radiographic examination, extending from the left to the right side. Furthermore, extensive resorptive tooth lesions were observed bilaterally, extending to the level of the left second mandibular premolar tooth. A leftward-deviated space-occupying mass was identified along the distal aspect of the left incisors. The space-occupying mass resulted in widening of the interdental space between the left first and second incisors and was associated with marked root resorption, particularly involving the left first incisor and left canine teeth (Figure 1B). Tissue samples were obtained via punch biopsy and subjected to histopathological evaluation.

Computed tomography (CT) revealed a mandibular mass measuring approximately 20 × 19 × 31 mm in diameter and associated with extensive osteolysis (Figure 1C). The osteolytic changes extended caudally to a point approximately 0.5 cm mesial to the left second premolar tooth (Figure 1D). The mass was extending to the level of the apex of right canine tooth (Figure 1E). The lesion was poorly demarcated from the surrounding soft tissue and exhibited heterogeneous contrast enhancement without a distinct capsule (Figure 1F). The left mandibular and retropharyngeal lymph nodes were asymmetrically enlarged compared with the contralateral side, suggesting regional lymphadenopathy. Three small nodular opacities measuring less than 2 mm in diameter were identified in the left cranial lung lobe. These findings were compatible with early pulmonary metastatic lesions, although differential diagnoses such as pulmonary osseous metaplasia or focal atelectasis could not be excluded. Mild enlargement of the colonic lymph nodes was observed.

**Figure 2:**
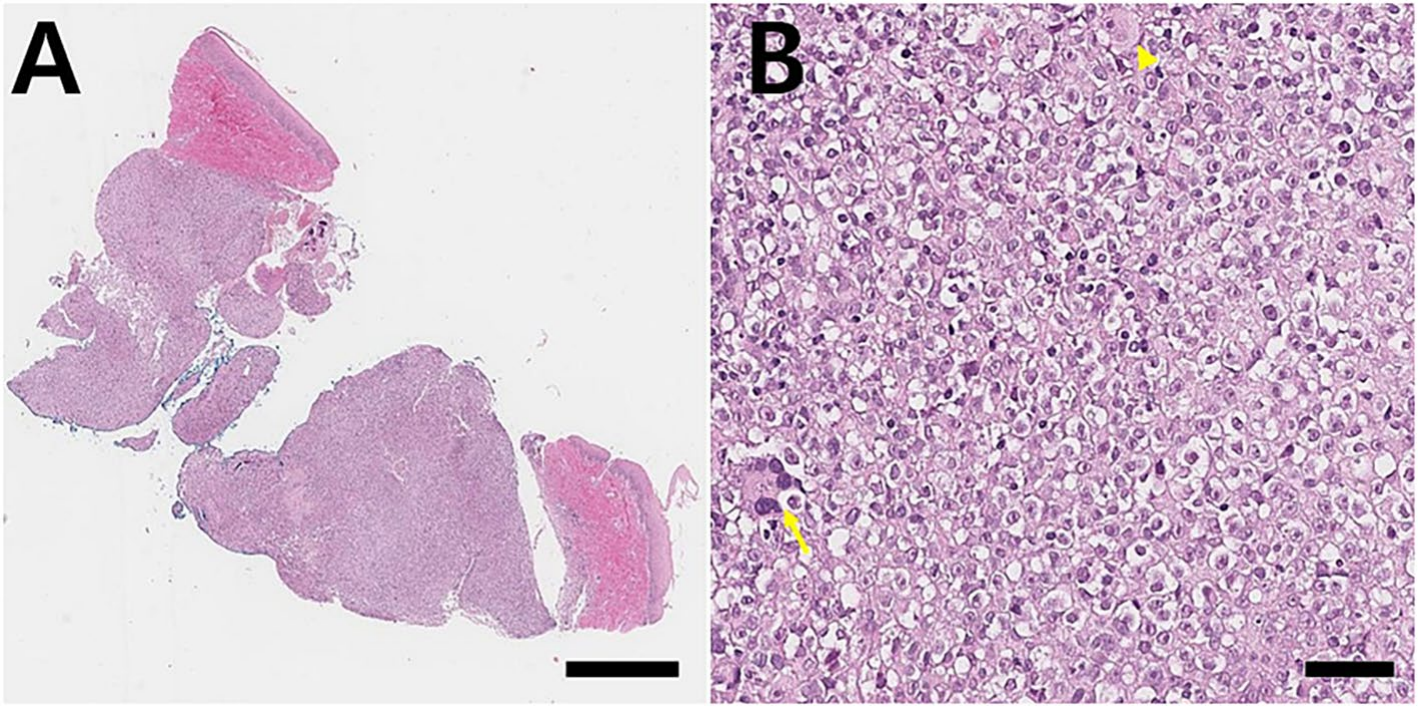
Histopathological findings of the gingival mass. **(A)** Low-power view (1.25× magnification) showing a densely cellular, poorly demarcated neoplastic proliferation expanding the lamina propria of the gingival mucosa. Hematoxylin and eosin stain; scale bar = 1 mm. **(B)** High-power view (20× magnification) demonstrating a population of round to polygonal neoplastic cells with moderate anisocytosis and anisokaryosis. A subset of neoplastic cells with indented nuclei and pale eosinophilic vacuolated cytoplasm, suggestive of histiocytic differentiation, is present (arrowhead). A multinucleated giant cell is also identified (arrow). Hematoxylin and eosin stain; scale bar = 50 μm.

**Figure 3:**
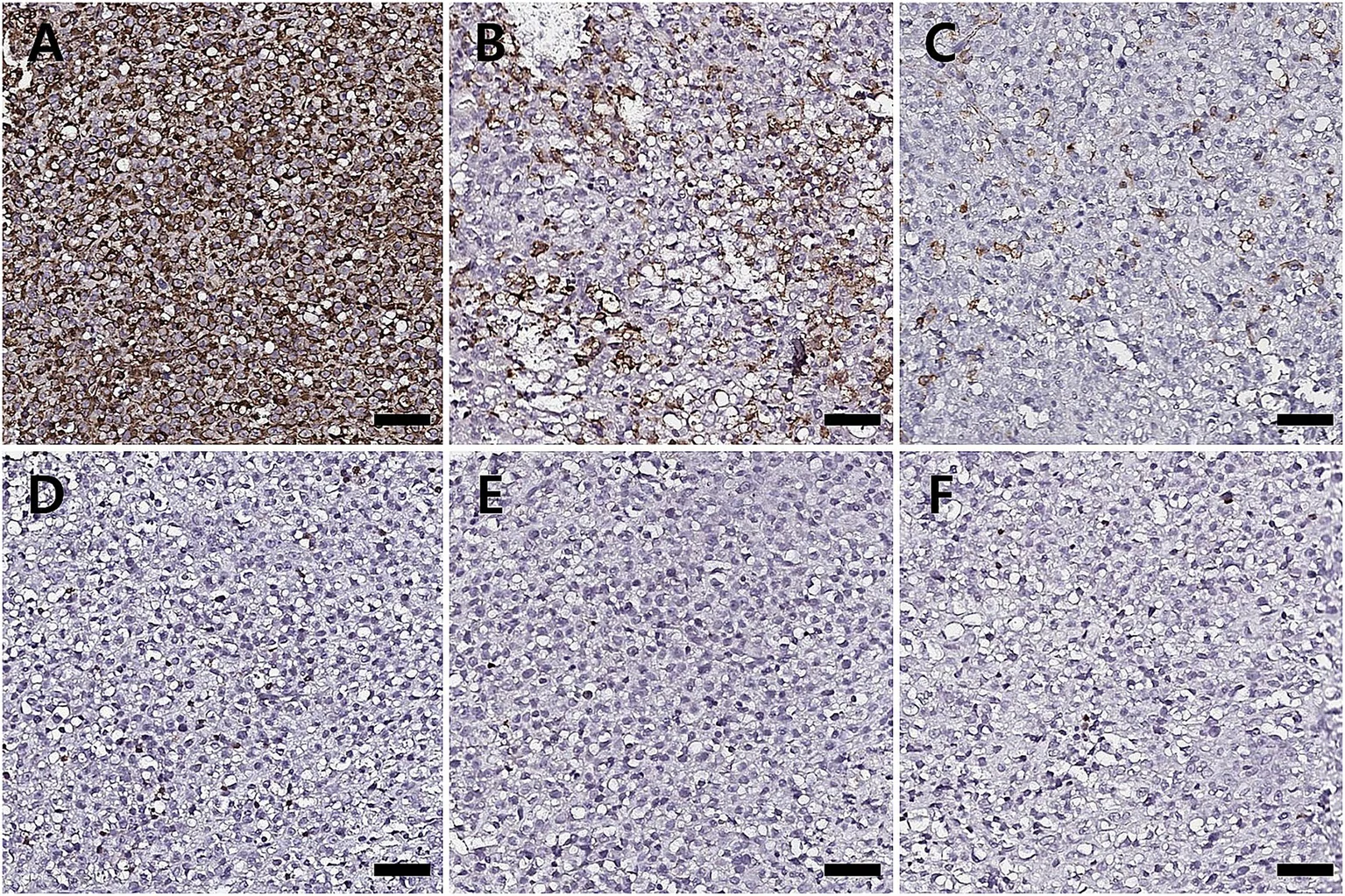
Immunohistochemical findings of the gingival mass. **(A)** Vimentin showing strong diffuse positivity throughout the neoplastic cell population. **(B)** Iba-1 demonstrating multifocal cytoplasmic positivity within a subset of neoplastic cells. **(C)** CD204 showing limited labeling in a smaller proportion of neoplastic cells. **(D)** CD3 immunolabeling restricted to scattered small lymphocytes within the tumor stroma; neoplastic cells are negative. **(E)** PAX5 negative in neoplastic cells. **(F)** MUM-1 negative in neoplastic cells. All images at 20x magnification. Scale bars = 50 μm.

Histopathological examination revealed that the lamina propria was expanded and infiltrated by a densely cellular, poorly demarcated population of neoplastic cells arranged in sheets, clusters, and packets within the resident collagenous stroma (Figure 2A). The neoplastic cells were predominantly round to polygonal, with variably distinct cell borders, and contained small to moderate amounts of lacy to vacuolated pale eosinophilic cytoplasm. The nuclei were round to ovoid and centrally located with one prominent nucleolus. Among the predominant round cell population, occasional neoplastic cells with indented nuclei and more abundant pale cytoplasm were identified, a morphological feature suggestive of histiocytic differentiation (Figure 2B). Moderate anisocytosis and anisokaryosis were observed with occasional multinucleated giant cells within the mass (Figure 2B). The mitotic count was 8 per 2.37 mm^2^. No lymphatic or vascular invasion was observed. Immunohistochemical evaluation was performed to determine the cell lineage (Figure 3). Vimentin showed strong diffuse positivity in the neoplastic cells, supporting their mesenchymal origin (Figure 3A). Iba-1 demonstrated multifocal cytoplasmic positivity within a subset of neoplastic cells (Figure 3B), whereas CD204 showed limited labeling in a smaller proportion of neoplastic cells (Figure 3C). CD3 immunolabeling was restricted to scattered small lymphocytes within the tumor stroma, with no reactivity detected in the neoplastic cells (Figure 3D). PAX5 and MUM-1 were negative, excluding the B-cell lymphoid and plasmacytic lineages (Figures 3E,F). Cytokeratin, Melan-A, and CD117 were negative, excluding cells of epithelial, melanocytic, and mast cell origins (Supplementary Data 1).

After obtaining consent from the owner, surgical removal of the mass by bilateral rostral mandibulectomy, extending from the right mandibular canine tooth to the left fourth premolar, was performed based on the CT results and histopathologic evaluation. As a premedication, butorphanol (0.2 mg/kg, intravenous [iv], Butophan^®^, Myungmoon Pharmaceutical, Seoul, Korea), cefazolin (30 mg/kg, iv, Chongkundang, Seoul, South Korea), and omeprazole (0.5 mg/kg, iv, Nexium^®^, AstraZeneca, London, UK) were administered. After injection of alfaxalone (4 mg/kg, iv, Alfaxan®, Jurox, Australia), anesthesia was maintained with isoflurane (Isoflurane^®^, Choongwae Pharmaceutical, Seoul, Korea). A constant-rate infusion (10 mL/kg/h) of Hartmann’s solution (HARTMANN’S sol^®^, Choongwae Pharmaceutical, Seoul, Korea) was administered during surgery.

**Figure 4:**
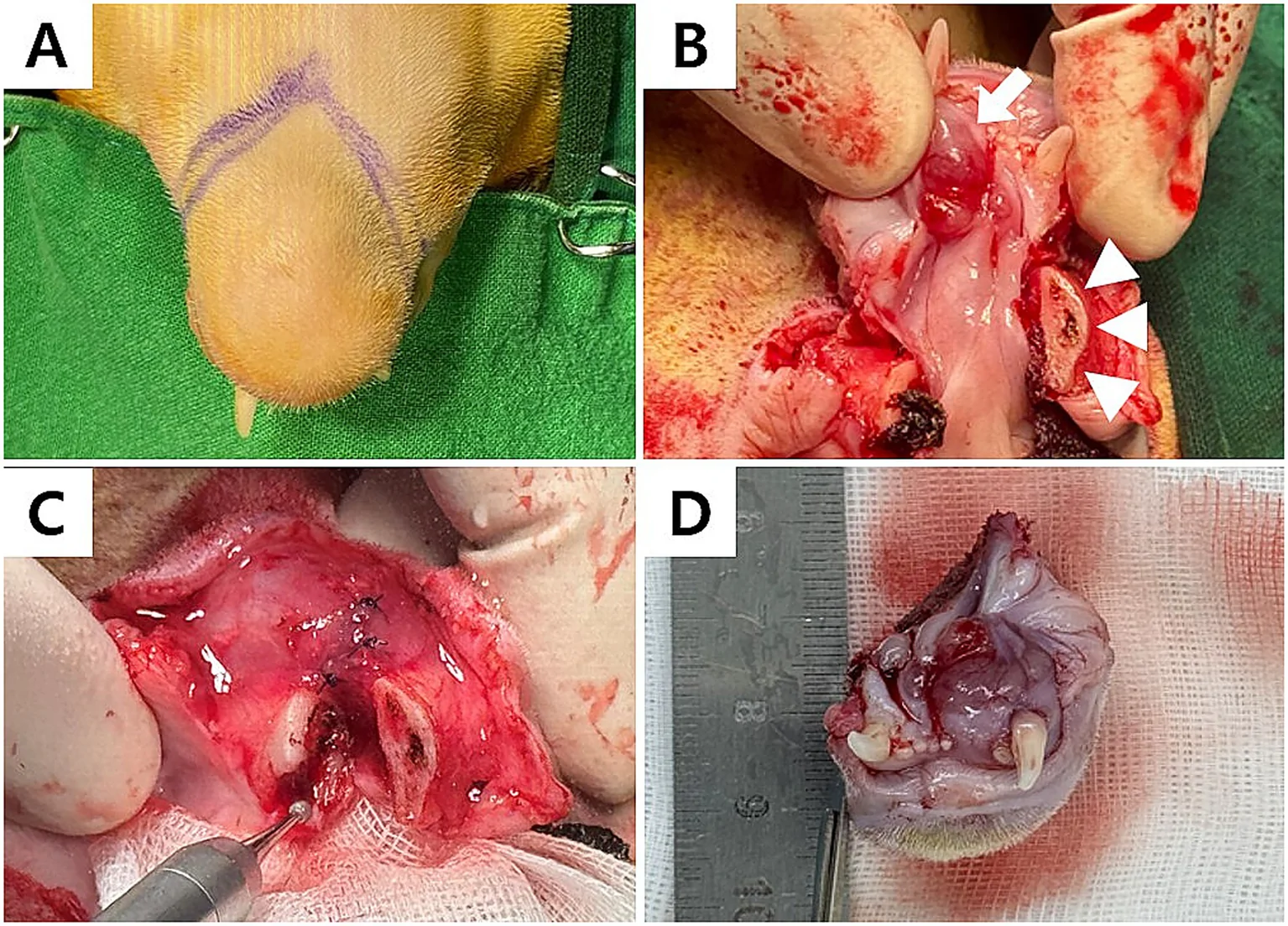
Intraoperative images. **(A)** The planned incision line is marked with a surgical skin marker along the ventral aspect of the mandible. **(B)** A mass with grossly normal tissue after bilateral mandibular osteotomy. **(C)** The sharp bony edges of the osteotomized mandibular segments were smoothed using a surgical burr. **(D)** Excised mass involving the rostral mandible with adjacent bilateral canine and incisor teeth.

Following general anesthesia, the patient was positioned in dorsal recumbency with the neck extended, and the surgical site was aseptically prepared. A modified V-shaped incision was made on the skin (Figure 4A). Bilateral rostral mandibulectomy was performed with surgical margins involving approximately 0.5 cm of grossly normal tissues, external to the grossly identifiable tumor margins. The osteotomy was performed approximately 5 mm distal to the right mandibular canine tooth, providing an approximately 5 mm gross surgical margin from the visible tumor. On the left side, the osteotomy was performed immediately mesial to the left mandibular second premolar tooth, based on preoperative CT planning, to achieve an approximately 5 mm gross surgical margin from the tumor. (Figure 4B). After osteotomy, the inferior alveolar neurovascular bundles were transected after the vessels were doubly ligated with 4–0 polydioxanone (PDS). The salivary ducts were similarly ligated with PDS 4–0 and transected. The soft tissues, including the tumor mass, were excised en bloc while preserving the tongue base. After bone transection, additional hemostasis was achieved with Surgicel^®^, which was removed prior to closure once bleeding was controlled. Sharp bony edges were smoothed using a surgical burr (Synthes EPD Burr; DePuy Synthes, Oberdorf, Switzerland) (Figure 4C). The remaining mandibular musculature on both sides was apposed across the defect with PDS 4–0 to reconstruct the soft tissue defect. The oral mucosa was closed with 5–0 PDS and a V-shaped skin incision was closed routinely using 4–0 PDS for the subcutaneous tissues and 4–0 nylon for the skin. An esophageal feeding tube was placed to provide postoperative nutritional support. The patient recovered from anesthesia without any complications. For postoperative pain management meloxicam (0.1 mg/kg, subcutaneous, Vetrocam^®^, Green Cross Veterinary Products, Yongin, South Korea) was administered once daily, and tramadol (3 mg/kg, iv, Maritrol^®^, Jeil Pharmaceutical, Daegu, South Korea) were administered twice daily.

The excised tumor tissue (Figure 4D) was submitted fresh for chemotherapeutic sensitivity testing (PODO Therapeutics, Seongnam, Gyeonggido, South Korea); therefore, histopathologic assessment of the final surgical margins was not performed. The patient was discharged 6 days after surgery. After confirming spontaneous appetite, the esophagostomy tube was removed on postoperative day 11. The chemotherapeutic sensitivity test revealed that mitoxantrone and doxorubicin were effective against the tumor. However, due to the owner’s financial constraints, these agents were not administered postoperatively.

Follow-up monitoring of the enlarged colonic lymph node and pulmonary lesions were recommended. However, further communication with the owner was unsuccessful. The patient presented to the hospital 7 months after surgery with lethargy and anorexia. Thoracic radiographs revealed multiple soft-tissue opacity masses distributed throughout both lung lobes. Abdominal ultrasonography identified multiple hyperechoic masses in the liver and kidneys, raising suspicion for metastasis. No recurrence of the histiocytic sarcoma was detected at the primary surgical site. With the owner’s consent, the patient was euthanized.

## 3 Discussion

This case represents a mandibular gingival histiocytic sarcoma in a 6-year-old Ragdoll cat and demonstrates several notable clinical differences from the two previously reported cases. In contrast to those reports, which involved 15- and 11-year-old cats, respectively, the present case occurred in a substantially younger animal (6, 7). Given that oral malignancies in cats are most frequently encountered in geriatric individuals, the present case raises the possibility that oral HS may develop over a wider age range.

Histopathologically, the neoplasm was highly cellular, composed predominantly of round cells with a minor spindle cell component arranged in sheets, exhibiting marked cellular atypia, multinucleated giant cells, and a high mitotic index (8 per 2.37 mm^2^). This mitotic count was markedly higher than that reported in a previously described case (1 per 10 HPF) (6), and comparable to that reported in another study (10 per 10 HPF) (7), supporting the relatively high proliferative activity observed in the present case. On immunohistochemical evaluation, strong diffuse vimentin positivity confirmed a mesenchymal origin, whereas negative results for CD3, PAX5, MUM-1, CD117, cytokeratin, and Melan-A excluded lymphoid, plasmacytic, mast cell, epithelial, and melanocytic lineages. Iba-1 and CD204 demonstrated partial positivity within a subset of neoplastic cells, supporting the histiocytic differentiation of the tumor. Limited or variable immunolabeling of both Iba-1 and CD204 has been described in previously reported cases of feline HS. With respect to Iba-1, staining was characterized as scattered, rare, and weak within neoplastic cells in one report (6) and as mild and multifocal in a case of feline intestinal histiocytic sarcoma (11). Regarding CD204, its immunoreactivity has also been shown to vary among feline HS cases, with some tumors demonstrating the iDC/macrophage immunophenotype (CD204+/E-cadherin−) while others showed negative or heterogeneous expression (12). Limited expression of histiocytic markers in the present case may reflect the poorly differentiated nature of the tumor. Overall, the morphological findings and results of multiple immunohistochemical stains supported the diagnosis of a poorly differentiated malignant mesenchymal neoplasm with histiocytic differentiation, favoring histiocytic sarcoma.

In the present case, bilateral rostral mandibulectomy was performed based on the CT findings and preoperative assessment, with resection extending into grossly normal tissues beyond the visible tumor margins. Two previously reported feline oral HS cases showed less favorable clinical courses (6, 7). Necova et al. reported a histiocytic sarcoma arising from the caudal soft palate, which was managed surgically at the cytoreductive level. Because the resection was incomplete owing to the location of the mass and the potential for metastasis, adjuvant chemotherapy was administered for 2–3 weeks. However, it was discontinued owing to recurrent pancreatitis. The patient was euthanized 6 months after surgery (6). On the other hand, Ruppert et al. described a histiocytic sarcoma involving the rostral mandible extending from the gingiva to the mucocutaneous junction. The patient elected palliative care because of the potential complications and poor long-term survival rates associated with mandibulectomy in cats. The patient was euthanized 6 months after the owner’s initial mass observation (7).

In contrast, in the present case, the cat remained free of recurrence at the primary surgical site for 7 months after surgery without adjuvant chemotherapy. Although a gross surgical margin of approximately 0.5 cm was planned rather than a conventionally wide margin, preoperative CT evaluation enabled careful assessment of the extent of mandibular invasion and surrounding soft tissue involvement. Based on the preoperative CT findings, the osteotomy was planned to include approximately 0.5 cm of grossly normal tissue beyond the visible tumor margins while achieving complete gross excision. Although histologically clean margins could not be confirmed because the specimen was submitted for chemosensitivity testing, no local recurrence was identified at the primary surgical site during follow-up. Despite the successful local control in the present case, wide surgical excision with histologically clean margins is regarded as a major determinant of local tumor control and long-term outcome in soft tissue sarcomas (13), and, similarly, margin status has been associated with both progression-free and overall survival in cats with oral tumors (14).

The lack of local recurrence at the primary surgical site in this case may have been associated with the extent of surgical resection and the relatively young age of the cat at diagnosis. However, the presence of metastatic lesions at the time of the patient’s re-presentation 7 months after surgery, were identified. Given the presence of a suspected metastatic lesion in the left cranial lung lobe and enlargement of the colonic lymph node on preoperative computed tomography, it is reasonable that the masses identified in the lungs, liver, and kidneys at the 7-month postoperative follow-up, which were highly suggestive of metastatic disease, represented progression of distant metastatic lesions that had already been established before surgery. To the best of our knowledge, this is the first reported case of histiocytic sarcoma arising from the oral mucosa managed by bilateral rostral mandibulectomy in a Ragdoll cat.

Although the overall findings favored the diagnosis of histiocytic sarcoma, several limitations should be considered. First, CD18 and MHC II were not included in the immunohistochemical panel, because these antibodies were not locally available at the time of diagnosis. In previously reported cases of feline oral HS, both markers demonstrated broader and more consistent reactivity than Iba-1. In one case, Iba-1 labeling was scattered and weak, yet CD18 and HLA-DR showed strong reactivity throughout the neoplastic cell population (6). In another study, Iba-1 immunolabeling was mild and multifocal, whereas MHC II and CD204 labeled a substantially greater proportion of tumor cells (11). Nonetheless, the diagnosis in the present case was based on the combined interpretation of the morphological features, partial Iba-1 and CD204 immunoreactivity, and the results of multiple other immunohistochemical stains. Second, histopathological assessment of the final surgical margins was not performed because the excised tumor tissue was submitted fresh for chemotherapeutic sensitivity testing. Third, although no recurrence developed at the primary surgical site during the seven-month postoperative period, the patient developed multiple lesions highly suspicious for metastatic disease and was euthanized. Therefore, this case demonstrates favorable local control after surgery but does not allow conclusions regarding long-term survival or systemic disease control.

## 4 Conclusion

Feline oral histiocytic sarcomas are extremely rare, and the small number of reported cases limits our understanding of their biological behavior, diagnosis, and clinical course. The present case expands on the available literature by documenting this tumor in a relatively young cat and demonstrating favorable short-term outcomes after surgical resection. Although oral histiocytic sarcoma remains an uncommon differential diagnosis, it should still be considered in cats with destructive oral masses, even in younger animals. Further case studies are required to clarify the prognostic range and establish evidence-based guidelines for clinical management.

## Data Availability

The original contributions presented in the study are included in the article/Supplementary material, further inquiries can be directed to the corresponding author/s.
